# The Neglected Nuclei

**DOI:** 10.3390/molecules26102982

**Published:** 2021-05-18

**Authors:** Peter Politzer, Jane S. Murray

**Affiliations:** Department of Chemistry, University of New Orleans, New Orleans, LA 70148, USA; jane.s.murray@gmail.com

**Keywords:** electrostatic potentials, electronic density donation and withdrawal, electrostatic potentials at nuclei, through-space effects of nuclei, polarization, nuclear potentials, atomic and molecular energy relationships, dispersion interactions

## Abstract

Since the nuclei in a molecule are treated as stationary, it is perhaps natural that interpretations of molecular properties and reactivity have focused primarily upon the electronic density distribution. The role of the nuclei has generally received little explicit consideration. Our objective has been to at least partially redress this imbalance in emphasis. We discuss a number of examples in which the nuclei play the determining role with respect to molecular properties and reactive behavior. It follows that conventional interpretations based solely upon electronic densities and donating or withdrawing tendencies should be made with caution.

## 1. The Born–Oppenheimer Approximation

A mainstay of quantum chemistry is the Born–Oppenheimer approximation [1]. This is based upon the fact that the electrons of a system move much more rapidly than the nuclei, which are considerably heavier. The electrons can essentially instantaneously adjust to nuclear motion and thus it is generally a very good approximation to decouple the nuclear and electronic motions and to treat the electrons as moving among stationary nuclei.

It is worth noting that the significance of the Born–Oppenheimer approximation is not limited to facilitating computations. As pointed out by Cramer [2], it makes possible the concepts of potential energy surfaces, and thus equilibrium and transition states.

With the Born–Oppenheimer approximation, the focus is upon obtaining the *electronic* wave function of a system, so it is not surprising that the emphasis in analyzing the properties and reactivities of molecules has been upon electronic effects. The important roles of the nuclei have received much less attention.

Our objective in this paper is to take a step towards remedying this situation. We will present a series of examples that explicitly demonstrate the significant influence that the nuclei can have upon atomic and molecular properties and reactive behavior. This will at the same time suggest caution in conventional interpretations that emphasize substituent and heteroatom electronic donating and accepting tendencies.

Atoms and molecules interact through their electrostatic potentials and the resulting electric fields. We will accordingly begin with a discussion of these.

## 2. Electrostatic Potentials and Electric Fields

The nuclei and electrons of any atom or molecule create an electrostatic potential V(**r**) at every point **r** in the surrounding space, given rigorously by Equation (1):

(1) $$V(r)=\sum_{A}\frac{Z_{A}}{\left|R_{A}-r\right|}-\int \frac{\rho (r^{\prime })dr^{\prime }}{\left|r^{\prime }-r\right|}$$

Z_A_ is the charge on nucleus A, located at **R_A_**, and ρ(**r**) is the electronic density of the atom or molecule.

The sign of V(**r**) in any given region of space depends upon whether the positive contributions of the nuclei or the negative ones of the electrons are dominant there. Positive regions will tend to interact favorably with nucleophiles (negative sites), and negative regions with electrophiles (positive sites).

The electrostatic potential is a real physical property, and is observable. It can be determined experimentally, by diffraction methods [3,4,5], as well as computationally.

In analyzing atomic and molecular interactions, V(**r**) is now generally computed on the surface of the atom or molecule, defined as an outer contour of its electronic density [6], typically the 0.001 au contour. The potential on this surface is labeled V_S_(**r**), and its locally most positive and most negative values, of which there may be several, are designated as V_S,max_ and V_S,min_, respectively.

An electrostatic potential V(**r**) creates an electric field **ε**(**r**) equal to the negative gradient of V(**r**), Equation (2):

(2) ε(r)=−∇V(r)

This electric field exerts a polarizing force upon any other nearby atom or molecule, thereby distorting to some extent the charge distribution of that atom or molecule from what it was in the unperturbed ground state. Since an electric field always accompanies an electrostatic potential, it is in principle never valid to try to separate electrostatics and polarization (although this is frequently done). The degree of polarization may sometimes be quite small, and neglecting it may be a reasonable *approximation* in particular cases, but it is always present [7,8,9].

## 3. Methods

All calculations were at the density functional M06-2X/6-31+G(d,p) level. Gaussian 09 was used for geometry optimizations and energies [10] and the WFA-SAS code for electrostatic potentials [11].

## 4. Free Neutral Atoms

We will begin our discussion of nuclear effects by considering free neutral atoms. The electronic density of a neutral atom is, on average, spherically symmetrical [12]. It has been shown that the electrostatic potential monotonically decreases from the nucleus, but is *positive everywhere* [13], even though the atom is overall neutral. Evidently, the concentrated positive charge of the nucleus dominates over the dispersed negative charges of the electrons.

The fact that V(**r**) of a neutral atom is positive everywhere fully supports Feynman’s explanation of the dispersion interaction between two atoms A and B [14]. As the atoms approach each other, the electronic distribution of each becomes slightly polarized toward the other. This happens because the positive electrostatic potential of B creates an electric field that exerts an attractive force upon the electronic charge of A, and polarizes a small amount of it into the internuclear region. Similarly, the force due to the electric field produced by the positive electrostatic potential of A polarizes a small amount of the electronic charge of B into the internuclear region.

The subsequent attraction of each nucleus for *its own* polarized electronic charge is what keeps the two atoms “bonded” together. Feynman’s concept has been verified by others on several occasions [15,16,17], and it has been shown to reproduce the 1/R^6^ dependence of dispersion interaction energies.

**Figure 1:**
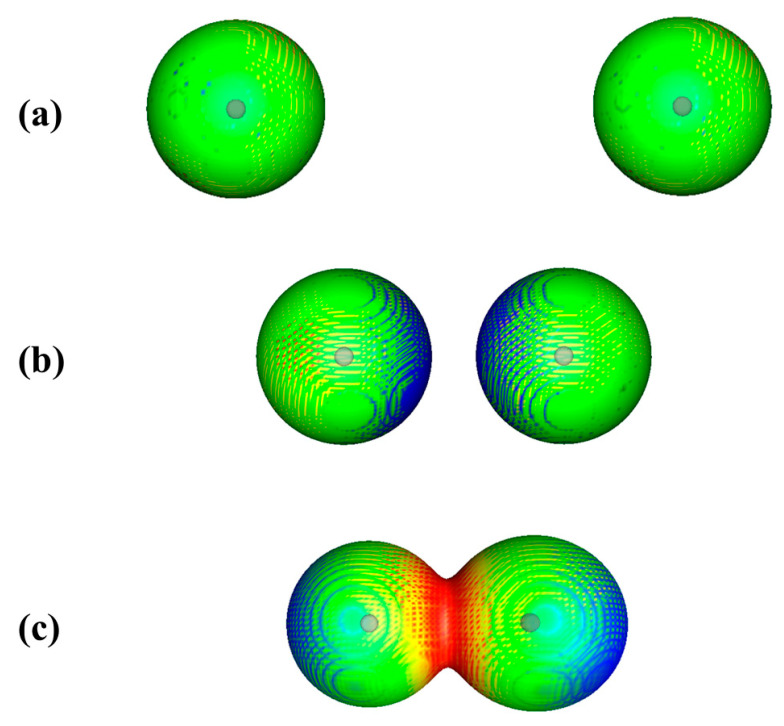
Computed electrostatic potentials on 0.001 au surfaces of two approaching argon atoms. Circles indicate positions of nuclei. Separations are (**a**) 10.0, (**b**) 5.0 and (**c**) 3.758 Ǻ (experimental equilibrium distance). Color ranges, in kcal/mol, are—red, more positive than 2.0; yellow, between 2.0 and 1.9; green, between 1.9 and 1.8; blue, between 1.8 and zero.

The polarization is demonstrated in Figure 1 for two approaching argon atoms. The experimental equilibrium internuclear distance in Ar_2_ is 3.758 Ǻ [18]. Figure 1a shows the two atoms prior to interaction, at a separation of 10.0 Ǻ. The 0.001 au contour of each atom’s electronic density is spherically symmetrical and V_S_(**r**) on this contour is uniformly positive.

In Figure 1b, the atoms are 5.0 Ǻ apart and beginning to interact. The regions facing each other are now slightly less positive than the rest of the surfaces, due to the slight polarization of each atom’s electronic density toward the other, as predicted by Feynman.

At the equilibrium separation of 3.758 Ǻ, Figure 1c, the 0.001 au contours of the two atoms overlap, indicating a small buildup of electronic charge in the internuclear region. Perhaps surprisingly, however, the electrostatic potential in the internuclear region is now even more positive than in Figure 1a. This is because the two nuclei are in relatively close proximity and their combined contributions to the internuclear electrostatic potential dominate over that of the internuclear electronic charge buildup. At the same time, the induced asymmetry of each atom’s charge distribution results in the outer region of each atom becoming slightly less positive.

What is significant is that V_S_(**r**) in the internuclear region becomes more positive *despite the buildup of electronic charge in that region*. This is an initial example of an important point that will be discussed in the next section.

## 5. The Electrostatic Potential Does Not Necessarily Follow the Electronic Density

The electrostatic potential at any given point is the result of the contributions of all of the electrons *plus* all of the nuclei of a system [19]. This means that V(**r**) does not simply follow the electronic density, as is often assumed in the literature [20,21,22,23,24]. Specifically, while “electron-rich” regions do frequently have negative electrostatic potentials, this is not necessarily the case. This has been particularly emphasized by Wheeler and Houk [25,26]; see also Politzer and Murray [27].

**Figure 2:**
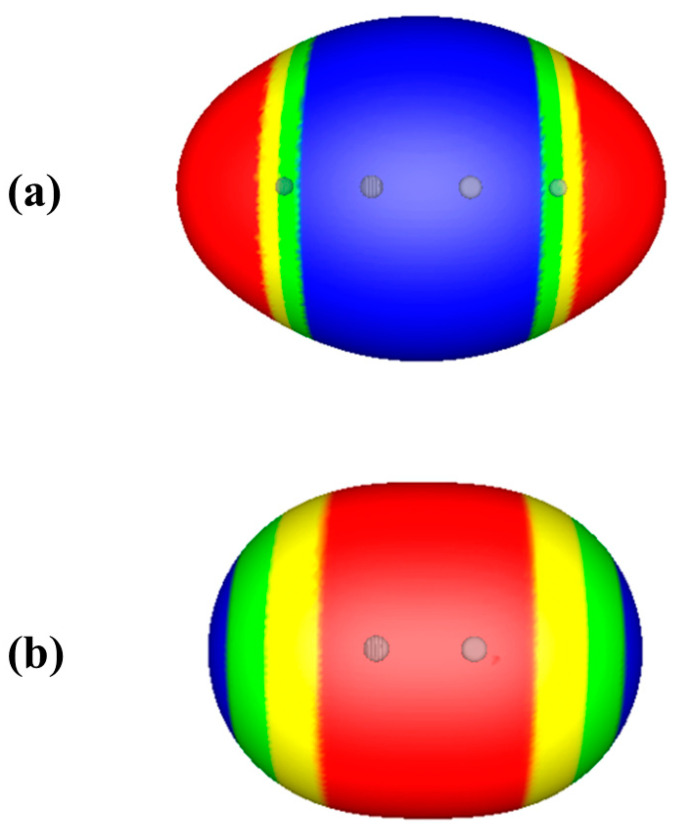
Computed electrostatic potentials on 0.001 au surfaces of (**a**) acetylene, HC≡CH, and (**b**) nitrogen, N≡N. Circles indicate positions of nuclei. Color ranges, in kcal/mol, are—red, more positive than 4; yellow, between 4 and zero; green, between zero and −4; blue, more negative than −4.

Consider the isoelectronic molecules acetylene and nitrogen, HC≡CH and N≡N. Formally, both have triple bonds, so both can be described as electron-rich in the C≡C and N≡N internuclear regions. However, Figure 2 shows that while the electrostatic potential on the 0.001 au surface of acetylene is completely negative in the C≡C region, as expected, V_S_(**r**) of the nitrogen molecule is completely positive in the N≡N region. This reflects the fact that the nuclei closest to the triple bond region in N≡N have more positive charges, +7, than those in HC≡CH, +6.

**Figure 3:**
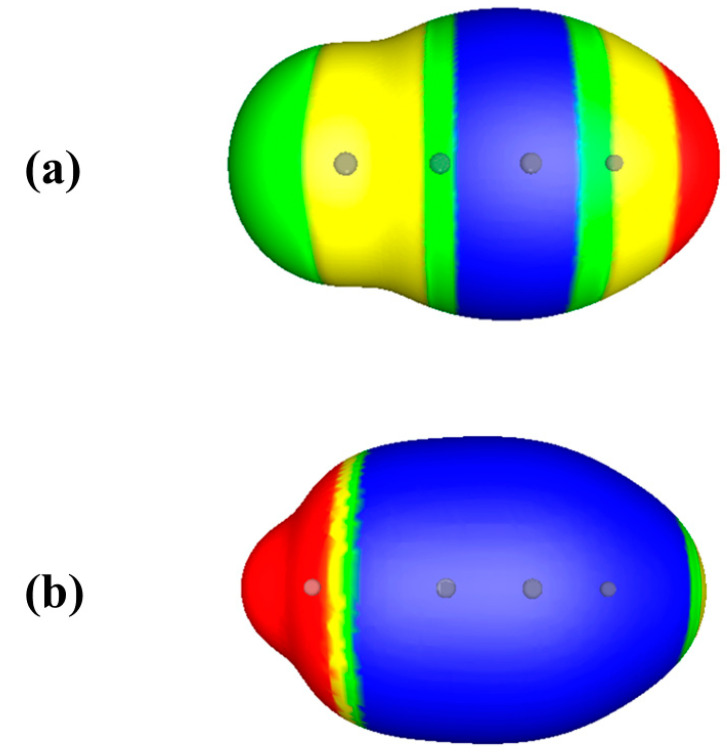
Computed electrostatic potentials on 0.001 au surfaces of (**a**) fluoroacetylene, FC≡CH, and (**b**) lithium acetylene, LiC≡CH. Circles indicate positions of nuclei. Fluorine is at the left in (**a**), lithium is at the left in (**b**). Color ranges, in kcal/mol, are—red, more positive than 15; yellow, between 15 and zero; green, between zero and −5; blue, more negative than −5.

Now consider two substituted acetylenes, FC≡CH and LiC≡CH. These do both have negative electrostatic potentials in the triple bond regions (Figure 3). The triple bond region of FC≡CH is the less negative, with V_S,min_ = −8.9 kcal/mol, compared to −41.3 kcal/mol for LiC≡CH. The conventional explanation for this is electron withdrawal by the more electronegative fluorine, and electron donation by the lithium.

However, the total amount of valence electronic charge in the triple bond regions of the two molecules is exactly the same—4.37 au [28]. If one looks only at the π electronic charge in these regions, FC≡CH actually has more, 2.45 au compared to 2.29 au for LiC≡CH, despite the electronegativity of fluorine being considerably greater than that of lithium. So why does FC≡CH have the less negative electrostatic potential in the triple bond region? And why is the potential around the highly electronegative fluorine actually positive? The obvious answer is the much more positive nuclear charge of fluorine, +9 vs. +3 for lithium.

**Figure 4:**
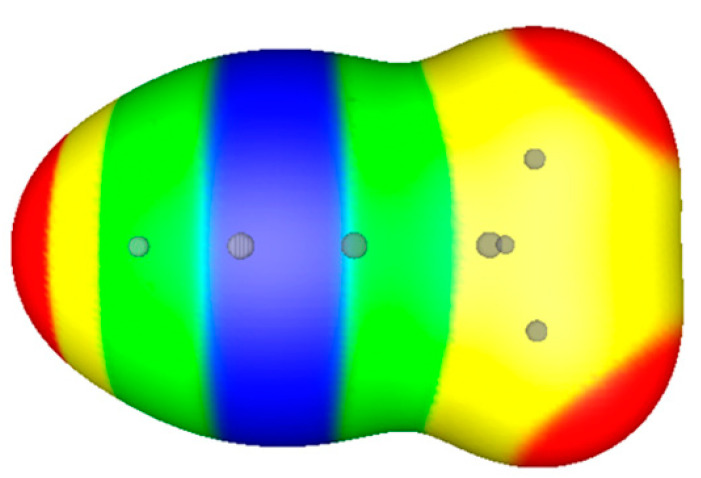
Computed electrostatic potential on 0.001 au surface of propyne, HC≡C-CH-CH_3_. Circles indicate positions of nuclei. The methyl group is at the right. Color ranges, in kcal/mol, are—red, more positive than 12; yellow, between 12 and zero; green, between zero and −15; blue, more negative than −15.

One more example of a substituted acetylene involves propyne, HC≡C-CH_3_. The electrostatic potential in the triple bond region is again negative, as seen in Figure 4, but more so around the unsubstituted carbon than the one bearing the methyl group. This means that electrophilic attack will be favored to occur on the unsubstituted carbon. This is in accord with Markownikoff’s rule, but seems to be inconsistent with the fact that a methyl group is considered to be weakly electron-donating [29], which suggests that V_S_(**r**) should be more negative around the methylated carbon. It is not, however, which can be attributed to the nearby carbon nucleus in the methyl group, with its +6 charge.

## 6. Through-Space Substituent Effects

From substituted acetylenes we move to substituted benzenes, and the extensive analyses of substituent effects upon their electrostatic potentials that have been carried out by Wheeler and Houk [25,26,30]. Benzene itself has negative V_S_(r) in the π regions above and below the ring [25]. The change in this that accompanies the introduction of substituents has conventionally been ascribed to the donation or withdrawal of electronic density by the substituents.

However, Wheeler and Houk drew attention, with supporting evidence, to the role of “through-space” contributions by the nuclei and electrons of the substituents to the electrostatic potentials in the π regions. These through-space effects are in addition to the donation or withdrawal of electronic density by the substituents, and may often be dominant.

We will illustrate this with the fluorinated benzenes. With the introduction of successive fluorines, the electrostatic potential above and below the benzene ring becomes progressively less negative until, for 1,3,5-trifluorobenzene, it is completely positive and becomes increasingly more positive in continuing to hexafluorobenzene [31].

This progression from negative to positive π regions is traditionally attributed to withdrawal of electronic density by the highly electronegative fluorines. However, Wheeler and Houk showed that the primary cause is largely the through-space effect of replacing +1 hydrogen nuclei by +9 fluorine nuclei; withdrawal of electronic density is a lesser factor [25]. Note the parallels to what was discussed in Section 5 for substituted acetylenes; these were also through-space effects.

## 7. Nitrogen Heterocycles

Wheeler and Bloom extended the preceding analyses to some nitrogen heterocycles, specifically the azines **1**–**5** [32]. As mentioned above, the electrostatic potentials in the π regions of benzene are negative [25], but they become progressively more positive as carbons are replaced by nitrogens in going through the azines from **1** to **5** [32,33]. This has in the past been attributed to electron withdrawal by the electronegative nitrogens, producing “electron-deficient” π regions.


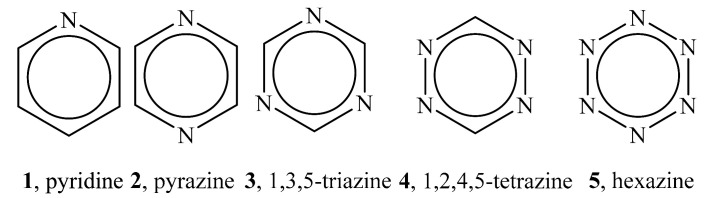


However, Wheeler and Bloom demonstrated that the increasingly positive V_S_(**r**) in the π regions are in fact the result of replacing carbons, with nuclear charges of +6, by nitrogens, having nuclear charges of +7 [32]. Thus, these are again examples of through-space effects. Wheeler and Bloom did report some changes in the π region electronic densities, but concluded that electron-deficient π regions are not responsible for the increasingly positive V_S_(**r**).

## 8. The Nuclear Potential

The term “nuclear potential” refers to the electrostatic potential ν(r) produced at a point **r** by just the nuclei of a molecule, Equation (3):

(3) $$\nu (r)=\sum_{A}\frac{Z_{A}}{\left|R_{A}-r\right|}$$

It is well known from the Hohenberg–Kohn theorem that the electronic density ρ(**r**) completely determines ν(r) and all other ground-state properties of the molecule [34]. Hohenberg and Kohn noted that, conversely, ρ(**r**) is a unique functional of ν(r), Equation (4):

(4) ρ(r)=f[ν(r)]

Parr and Yang have also pointed this out [35]. Equation (4) means that ν(r) can also be viewed as a determinant of the molecule’s ground-state properties; it certainly attests to the fundamental nature of the nuclear potential.

Parr and Berk [36] have investigated the nature of the relationship in Equation (4) and showed that, for many molecules (but not all [37]), the contours of ρ(**r**) are overall similar to those of ν(r) (see also Tal et al. [38]). This similarity led Parr and Berk to speculate that ν(r) could sometimes be used as a first approximation to ρ(**r**).

## 9. Energy Relationships

From the electrostatic potential created by the nuclei of a molecule at any point **r**, discussed in the previous section, we go now to the potential V_0,A_ created at the position of each nucleus A by the electrons and the other nuclei of a molecule. This is given by Equation (5):

(5) $$V_{0,A}=\sum_{B\neq A}\frac{Z_{B}}{\left|R_{B}-R_{A}\right|}-\int \frac{\rho (r)dr}{\left|r-R_{A}\right|}$$

**R**_A_ is again the position of nucleus A, having charge Z_A_, and **R**_B_ and Z_B_ are the position and charge of any other nucleus B. If A is the nucleus of a single free atom, then V_0,A_ is just the integral term in Equation (5) and is labeled V_0_.

An interesting feature of the electrostatic potential at the nucleus of an atom is how insensitive it is to the environment of the atom [39,40,41]. It typically varies by only 1–2% in molecules of different polarities, the free atom and even ions (the only exception is the hydrogen atom, which has only one electron and that electron is involved in bonding). The near-constancy of V_0,A_ for a given A is particularly remarkable because the two contributions to V_0,A_, the nuclear and electronic terms in Equation (5), can change considerably in going from one molecule to another. However, V_0,A_ changes very little and remains characteristic of the nucleus A.

Already two years before the Hohenberg–Kohn theorem, Wilson [42] had used the Hellmann–Feynman theorem [14,43] to derive an exact expression for the energy of a molecule in terms of its electronic density. This was subsequently re-formulated to give molecular energy rigorously as a function of the electrostatic potentials V_0,A_ at the molecule’s nuclei [44,45]:

(6) $$E_{\mathrm{mol}}=\sum_{A}z_{A}\int_{\lambda =0}^{1}{\left[V_{0,A}(\lambda )\right]}_{N}d\lambda$$

In Equation (6), N is the number of electrons and λ is a scaling parameter between zero and one such that the charge on any nucleus Z_i_ is λz_i_. In the actual molecule, λ = 1 and z_i_ = Z_i_ for each nucleus. The purpose of λ is to ensure that all of the nuclear charges increase in a concerted manner from zero to their true values.

The atomic version of Equation (6) is,

(7) $$E_{\mathrm{atom}}=\int_{Z^{\prime }=0}^{Z}{\left[V_{0}(Z\prime )\right]}_{N}\;dZ\prime$$

Equation (7) was introduced by Foldy [46] several years before Wilson’s expression for molecular energies.

Using Equations (6) and (7) to actually determine molecular and atomic energies is challenging, in part because the integrals need to be evaluated with the number of electrons held constant. However, the equations are very significant conceptually because they show that these energies, which are *two-electron properties*, can be expressed rigorously in terms of the electrostatic potentials at the nuclei, which are one-elecron properties. Evidently Equations (6) and (7) implicitly account for electron–electron repulsion. These relationships are certainly consistent with the Hohenberg–Kohn theorem.

It is further noteworthy that the molecular expression, Equation (6), is essentially the atomic expression, Equation (7), summed over all of the constituent nuclei. There are no explicit “mixing” terms, consistent with the concept of atoms in molecules.

Various approximate relationships between energies and electrostatic potentials at nuclei have also been proposed; some of these are modified forms of Equations (6) and (7). This work is discussed in several reviews [40,47,48].

Some approximate quantum chemical procedures may yield electrostatic potentials at nuclei more accurately than they do total energies. For instance, Hartree–Fock total energies are correct through first order, but Hartree–Fock V_0,A_ and V_0_ are correct through second-order [48,49,50]. Thus, if Hartree–Fock V_0,A_ or V_0_ are used in an approximate energy expression, the resulting energies may be better than Hartree–Fock, meaning that they may contain significant amounts of correlation energy [49,50,51], even though the Hartree–Fock energy does not include correlation. Levy et al. derived atomic energy expressions that gave nearly all of the correlation energies of the atoms H through Ar using Hartree–Fock V_0_ [49,52].

## 10. Discussion and Summary

Our objective in this paper has been to counteract, at least to some extent, the general tendency to focus solely upon electronic factors in interpreting atomic and molecular properties and reactive behavior. We have presented a series of examples to show the significant insights that can be gained by explicitly taking nuclear contributions into account. These are summarized as follows:

(1) Electrostatic potentials do not always follow the electronic density in a molecule. The potentials created by the nuclei must also be considered. Regions of high electronic density may have negative electrostatic potentials, but this is not necessarily the case;
(2) Because of the nuclear contributions, the electrostatic potentials in certain regions may sometimes contradict what would be anticipated from electronegativities and electron donating or withdrawing tendencies of substituents and heteroatoms. This does not invalidate these tendencies; it just means that nuclear effects need to be explicitly considered;
(3) The contours of the electronic density are frequently similar to the contours of the electrostatic potential due to the nuclei alone;
(4) The electrostatic potential at a nucleus that is created by the electronic density and the other nuclei of a molecule is characteristic of that nucleus and varies very little with the molecular, atomic or ionic environment of that nucleus; and
(5) Atomic and molecular energies can be expressed rigorously in terms of the electrostatic potentials at the nuclei of their constituent atoms. Electron–electron repulsion, including correlation, is taken into account *implicitly* rather than explicitly.

In conclusion, we emphasize again that the distribution of positive and negative electrostatic potentials in molecules should not be interpreted solely in terms of the electronic donating or withdrawing tendencies of substituents and heteroatoms. In particular, the through-space effects of the nuclear electrostatic potentials must be explicitly taken into consideration.

## Data Availability

Please contact the corresponding author.
